# Changes in Oral Health Policies and Guidelines During the COVID-19 Pandemic

**DOI:** 10.3389/froh.2021.668444

**Published:** 2021-05-20

**Authors:** Chloe Meng Jiang, Duangporn Duangthip, Prim Auychai, Mirei Chiba, Morenike Oluwatoyin Folayan, Hamdi Hosni Hamdan Hamama, Porawit Kamnoedboon, Karl Lyons, Oranart Matangkasombut, Kavita R. Mathu-Muju, Vijay Prakash Mathur, May Lei Mei, Mike Morgan, Suchit Poolthong, Morankar Rahul, Murali Srinivasan, Tetsu Takahashi, Sanicha Yaklai, Shinan Zhang, Xin Chun Zou, Chun Hung Chu, Edward Chin Man Lo

**Affiliations:** ^1^Faculty of Dentistry, The University of Hong Kong, Hong Kong, China; ^2^Faculty of Dentistry, Chulalongkorn University, Bangkok, Thailand; ^3^Graduate School of Dentistry, Tohoku University, Sendai, Japan; ^4^Obafemi Awolowo University, Ile-Ife, Nigeria; ^5^Faculty of Dentistry, Mansoura University, Mansoura, Egypt; ^6^Center of Dental Medicine, University of Zurich, Zurich, Switzerland; ^7^Faculty of Dentistry, University of Otago, Dunedin, New Zealand; ^8^Faculty of Dentistry, University of British Columbia, Vancouver, BC, Canada; ^9^Department of Pediatric and Preventive Dentistry, Centre for Dental Education and Research, All India Institute of Medical Sciences, New Delhi, India; ^10^Affiliated Stomatological Hospital, School of Stomatology, Kunming Medical University, Kunming, China

**Keywords:** oral health policy, oral health guidance, COVID-19, review, pandemic

## Abstract

The aim of this study was to describe the changes in oral health policies and guidelines in response to the Coronavirus disease 2019 (COVID-19) pandemic in different countries and regions around the world. Information on oral health policies and guidelines from 9 countries (Canada, China including Hong Kong, Egypt, India, Japan, New Zealand, Nigeria, Switzerland, and Thailand) were summarized, and sources of the information were mostly the national or regional health authorities and/or dental council/associations. The changes made to the oral health guidelines depended on the severity of the COVID-19 pandemic. This included suspension of non-emergency dental care services at the peak of the COVID-19 outbreak, and easing the restrictions on non-essential and elective dental care when the pandemic became under control. The COVID-19 risk mitigation strategies include strict adherence to infection control practices (use of hand sanitizers, facemask and maintaining social distancing), reducing the amount of aerosol production in the dental setting, and managing the quality of air in the dental treatment rooms by reducing the use of air conditioners and improving air exchange. The COVID-19 pandemic has shown a major impact on dental practice. Dental professionals are trying to adapt to the new norms, while the medium to long-term impact of COVID-19 on dentistry needs further investigation.

## Introduction

A newly identified pneumonia outbreak was first reported in China in December 2019. This was caused by a highly infectious pathogen named the severe acute respiratory coronavirus 2 (SAR-CoV-2). It has caused the global pandemic, coronavirus disease 2019 (COVID-19), which has affected 1% of the global population (79,231,893 cumulative cases up to 27 December 2020) and disrupted the world's usual socioeconomic daily activities [1]. As a result, managing and solving the COVID-19 crisis is currently a top government priority around the world.

The SARS-CoV-2 is transmitted through direct or indirect human-to-human contact via infected secretions such as respiratory and saliva droplets, or through contaminated surfaces [2]. The SARS-CoV-2 mainly affects the respiratory system of infected persons [3]. The transmission route is through close human contact (within 1–2 m) with infected individuals through mouth and nose secretion droplets [4]. The incubation period of the virus is usually between 5 and 6 days but can be up to 14 days [5]; thus, a quarantine period of around 2 weeks has been widely adopted in many countries. Common symptoms include cough, fever, and shortness of breath, which may result in respiratory failure and even death. Besides, fatigue, diarrhea, respiratory distress, unexplained muscle pain, sore throat, and loss of taste or smell have also been reported [6]. Patients with co-morbidities such as chronic renal, lung, and heart diseases are more likely to have severe or critical COVID-19 symptoms [3].

Apart from individual and public health problems, COVID-19 also challenges the world's economy. It was reported that the forecast gross domestic product in different countries would decline on average 5% [7]. Mitigation and suppression policies had significant economic impacts on dental practice [8]. Using a modeling approach, prevention (−80% in mean), periodontics (−76% in mean), and prosthetics (−70% in mean) would be the most affected utilization services [9]. As the mobility chain of human activities was disrupted, the demand for manufacturing and service industries' plummeted while the need for protection healthcare supplies was boosted. Uprising challenges such as high medical care costs and shortage of medical equipment and professionals are limiting the already fragile healthcare system from expanding to meet the current need [10]. Therefore, maintaining and balancing public healthcare services under such circumstances is crucial for governments worldwide.

Various public policies which involve issuing new laws, regulations, executive orders, local ordinances, and court decisions, have been established in different countries to control the pandemic [11, 12]. Many of these public health related policies such as the closure of borders, restrictions on intra-border travels, social distancing, work-from-home, school closures and economic lockdown have had a significant impact. Quarantine has averted 44–81% of new cases and lowered mortality by around 31–63% [13]. In addition, the economic support from state governments to fight the global pandemic has grown: the World Health Organization (WHO) for example, has raised over 200 billion US dollars for global strategic preparedness and humanitarian response plans [14].

Currently, there is very little information on the impact of the COVID-19 pandemic on the dental industry, which is one likely to be severely affected by the pandemic. The risk of transmission of SARS-CoV-2 via dental practice is relatively high due to exposure to saliva and blood [4]. In the US, a mitigating measure to reduce the risk of transmission of SARS-CoV-2 for both patients and oral health workers was the issuance of directives by the Centers for Disease Control and Prevention. The directives included that critical dental services should be prioritized and dental care be provided in an approach which minimizes the harm to patients from delaying dental treatment and harm to health personnel from potential exposure to SARS-CoV-2 infection [15]. Updated guidance for dental settings has been implemented in many countries around the world. Protocols on patient triage and workflow, and infection control guidelines on how to provide dental services during the pandemic have also been produced [16, 17]. Specific guidelines for different specialties such as orthodontics and endodontics have also been published [18, 19].

Though there have been multiple dental practice guidelines, protocols and directive developed for dental practice during the COVID-19 pandemic by dental regulatory boards and dental associations, little is known about how these have affected national and regional oral health policies. This review paper aimed to summarize the lessons learned from regions with different disease severity, and the policy implications of these lessons, and to suggest practical guides for reviewing national oral health policies that can inform safe oral healthcare practices beyond the pandemic.

## The Situation in Different Selected Countries/Regions

The COVID-19 pandemic is ongoing globally, with over 79 million confirmed cases and more than 1.7 million deaths reported to the WHO till December 2020 [14]. The updated situations of COVID-19 infection in different countries and regions as of 27 December 2020 are summarized in Table 1 [1]. Collaborators from each country/region independently collated the country level data on the oral health policy and dental practice guidelines in their countries.

**Table 1:** Updated situations of COVID-19 in different countries as of 27 December 2020 (by alphabetical order).

**Country**	**Cumulative confirmed cases**	**Cumulative cases per 1 million population**	**Cumulative deaths**	**Cumulative deaths per 1 million population**	**Cross infection from dental treatment**
Canada	539,298	14,289	14,781	392	Yes^*^
China (Hong Kong included)	96,324	65	4,777	3	No
Egypt	131,315	1,283	7,352	72	No
India	10,187,850	7,382	147,622	107	No
Japan	217,312	1,718	3,213	25	No
New Zealand	1,788	371	25	5	No
Nigeria	83,576	405	1,247	6	No
Switzerland	426,199	49,245	6,508	752	No
Thailand	6,020	86	60	1	No

*Data in this table was derived from the WHO, COVID-19 Weekly Epidemiological Update*.

* *October 14, 2020, outbreak in dental service setting in Ontario, Canada with 3 cases linked [20]*.

## Canada

Canada is the second largest country in the world in terms of land area and has 10 provinces and 3 territories. The 10 provinces exercise constitutional powers in their own right, while the territories exercise delegated powers under the authority of the Parliament of Canada (federal government). The regulation of health professionals in Canada falls under provincial jurisdiction, meaning that each province has its own legislation that affects dental services [21]. Dental regulatory bodies exist in every province and territory and are responsible for setting professional practice standards, including new measures required to mitigate the transmission of COVID-19 in the dental practice setting. Although dental professionals must follow the policies implemented by the dental regulatory authority in their regions, the new protocols tend to be largely uniform across the country [22–26]. The Canadian Dental Association (CDA), a non-regulatory authority, is a federation of Canada's provincial and territorial dental associations, and represents over 21,000 practicing dentists nation-wide. When dental offices across Canada were permitted to re-open, the CDA recommended that oral health care should not be delayed in order to promote good oral and overall health. The changes in the delivery of oral health care in Canada can be summarized as enhanced infection prevention and control procedures and the implementation of additional protective barriers and techniques to shield patients and dental personnel from potentially infectious agents.

There have been changes in patient management and treatment protocols in order to mitigate transmission of the virus. When patients call to schedule an appointment, screening questions are asked to determine a person's risk for COVID-19. Although screening tools vary somewhat by province, they typically include questions about any symptoms being experienced, travel history in recent weeks, and exposure to someone who has been confirmed to have COVID-19. Patients who screen or test positive and without urgent care need do not typically receive in-person care. Upon arrival at the appointment, the screening process is repeated and the individual's temperature is taken and recorded. Upon initial entry into the dental practice, patients are expected to complete hand hygiene with a 70–90% alcohol-based hand rub or soap and water. Patients wear facemasks until seated in the operatory and treatment is about to begin. Physical distancing of at least 2 m from other patients and staff is required, except for necessary patient care. During treatment, general recommendations include minimizing the use of the air/water syringe, punctilious use of the high volume evacuator and rubber dam, and a preference for extra-oral over intra-oral radiographs, if possible. Four-handed dentistry is required for the provision of all aerosol-generating procedures. Upon completion of treatment, patients are asked to don their facemasks. Contactless forms of payment are preferred, and distribution of office promotional materials is discouraged. Management of contact tracing varies by province. Typically, provincial health authorities are responsible for contact tracing in the event that an individual receives a positive COVID-19 test result within 14 days after the dental appointment [23–26].

Canada, with the exception of specific services, has a universal healthcare system that would provide necessary treatment for any health care providers exposed to COVID-19. The availability of testing varies among provinces; however, according to federal government modeling, Canada has a national capacity of ~200,000 tests per day. Personal protective equipment (PPE) requirements for the provision of dental treatment include donning a surgical cap, surgical gown, face shield and changing into scrubs in addition to level 3 surgical masks/N95 respirators and gloves. Dental office staff are required to wear PPE as suited to their role; because of the potential for contamination, all office personnel must change clothing or wear surgical gowns in the dental clinic setting. Dentists are required to review and update pandemic protocols with their staff. Physical distancing of at least 2 m is obligatory except as required to provide patient care. Staff must conduct regular hand hygiene, and shifts and breaks should be staggered to support physical distancing. COVID-19 screening protocols for all dental clinic staff are conducted daily, including recording of daily temperature. All Canadians who experience symptoms of COVID-19 are expected to stay home and inform the public health authority in their province or territory and obtain advice on what they should do, including whether a COVID-19 test is recommended [23–26].

National requirements for heating, ventilation, and air-conditioning (HVAC) systems in health care facilities are determined by the Canadian Standards Association. The rate of air exchange in a space is influenced by multiple factors, including wall and ceiling heights, the presence of windows that can be opened, and the physical layout of the clinic. The amount of time a room needs to be left fallow after aerosol-generating procedures varies depending on the rate of air exchange in the building. Therefore, dental regulatory authorities have encouraged their members to contact an HVAC specialist for a professional evaluation prior to changing existing clinic designs. It is appropriate to reduce aerosols at source with aerosol reduction techniques. Other changes in dental clinic facilities include limited numbers of patients in the waiting room, fabric surfaces replaced with easily disinfected furniture, removal of magazines and toys, and placement of Plexiglas barriers at the reception desk [23–26].

Private practices across Canada are adapting to the new guidelines. The degree to which this pandemic has affected each region in Canada varies immensely, and it is difficult to predict the long-term consequences on Canadian dental practices. Aside from emergency services, most dental procedures were considered non-essential when provinces and territories implemented initial pandemic restrictions. In the short term, the increased safety measures have come with added costs, supply chain issues, and less capacity to treat patients. According to the most recent Directive from the Chief Medical Officer of Health, dentists can now provide non-essential and elective care along with essential services, emergency and urgent care [23–26]. However, the enduring impact the pandemic will have on dentists and the practice of dentistry in Canada is not yet known.

## China

In late December 2019, cases of pneumonia of unknown cause were reported in Wuhan City, Hubei Province, China. The Wuhan City Health Commission issued an urgent notice on the diagnosis, treatment, prevention and control for this disease [27]. To better control the outbreak, the Chinese central government sent experts to Wuhan. The National Health Commission (NHC) of the People's Republic of China, along with the National Administration of Traditional Chinese Medicine and other related organizations have issued and updated diagnosis, treatment, prevention and control guidelines for COVID-19 [28]. As of 15 November 2020, the 8th edition of the guidelines were released [29].

At the end of January 2020, the NHC recommended that all health care workers use prevention precautions applied to Group A infectious diseases, such as cholera and plague [30]. As a result, the Chinese Stomatological Association (CSA) suggested that dental institutions should suspend routine dental treatments and only provide emergency dental treatment in public dental hospitals/departments [31]. Dentists were also required to follow strict standard precautions as well as additional preventive measures, such as wearing N95 facemask or equivalent, and isolation gowns. Aerosol-generating procedures were to be avoided or minimized [32]. During the COVID-19 outbreak, the CSA published recommendations for home-care methods of managing non-emergency oral diseases using social media and internet [33, 34] and many dental institutions started to provide online consultations to the public.

At the end of February 2020, the pandemic was gradually coming under control, and clinical dental services resumed nationwide. The recommendations on providing preventive care and treatment in dental practices were optimized with an increasing understanding of COVID-19 [35–37]. Generally speaking, a pre-check triage system was the vital stage for early screening and identification for suspected or confirmed cases. This phase was further modified as a population risk classification [37]. Patients are divided into three groups, high-, medium-, and low-risk then dental health care personnel (DHCP) follow one of three levels of personal protection, level 1 (standard), level 2 (advanced) or level 3 (strengthened protection). Irrespective of the level, patients are recommended to use antiseptic mouthrinse to gargle at the beginning of treatment [37]. During dental treatment, rubber dam and strong suction devices are used to minimize aerosol generation [35–37]. After treatment, DHCP remove their PPE in sequence and perform good hand hygiene following the Specification of Hand Hygiene for Healthcare Workers (WST313-2019) [36]. Dental instruments are reprocessed following the regulation for disinfection and sterilization technique of dental instruments (WS 506-2016) [36]. All high-frequency contact surfaces are disinfected, and the dental unit waterline is rinsed for 2 min with chlorinated water (100 mg/L). The treatment rooms are kept well-ventilated and the floor should be dry and clean, and disinfected every 2 h [36]. Medical waste, such as surgical masks and caps, are put in a designated area for temporary storage. These areas are cleaned and disinfected using chlorinated water (1,000 mg/L) [36].

## Egypt

In Egypt, practicing dentistry during the pandemic was challenging, due to the long lockdown period and patients' fear of being infected with the virus. Furthermore, there was concerning news of the first COVID-19 wave coming from Europe, in particular, Italy and the Mediterranean countries. This critical condition made practicing dentistry a very difficult task. In spite of the global shortage of PPE at the peak of COVID-19 pandemic from March to April 2020, most dental practitioners limited their clinical practice to emergencies and became on-call dentists.

Strict restorative protocols were implemented to minimize the risk for both patients and health workers. In the field of restorative dentistry, appropriate PPE and an aerosol-free working environment were needed to be maintained. Updating patient's medical history, including carefully assessing the medical history, has been essential before providing any restorative dental treatment. In addition, measuring body temperature and practicing in a well-ventilated clinic were recommended to minimize risk. The use of suction and air purifiers was also mandatory at this critical period. The application of rubber dam is recommended to reduce aerosol and cross-infection hazards.

Management of deep carious lesions has been the most challenging clinical task during the COVID-19 pandemic because of the risk to the patient of developing an irreversible pulpitis, although understanding current caries removal protocols helped overcome this problem in some cases. The selective caries removal concepts can maintain pulp vitality with minimal surgical intervention. This concept stated that “Any grossly softened caries-infected-dentine must be excavated, nevertheless, in deep carious lesions where the inner most layer of dentine, which directly covers the pulp, contains a high concentration of bacteria provided to create well-sealed restorations” [38]. Besides, non-aerosol generating procedures such as chemomechanical caries removal technique [39, 40] and silver diamine fluoride application [41] may be good for dental practice during the pandemic time.

## Hong Kong

Hong Kong is a highly dense city with over 7 million people. The COVID-19 pandemic has resulted in the infection of over 5,000 people in Hong Kong with more than 100 people deaths [42]. There has been community outbreak as many local cases cannot be traced to the identifiable sources, and some confirmed cases are asymptomatic. The government has tightened up various public health measures, such as mandatory wearing of facemasks in transportation and public indoors areas, limiting the number of people gathering in public, and temporary closure of bars and nightclubs.

In terms of dental care management in Hong Kong during the COVID-19 pandemic, the authorities have kept updating the situation of COVID-19 with dentists since January 2020 [43]. The government Department of Health strongly recommended dental professionals strictly adhere to the infection control principles, e.g., wear PPE properly and keep good hand hygiene and to avoid carrying out aerosol-generating procedures without the use of rubber dam. Certain procedures have been recommended to reduce aerosol generation and mitigate the potential contamination by the aerosol generated by using:

- pre-procedural mouth rinse with suitable antiseptic solution (such as povidone- iodine, hydrogen peroxide, chlorhexidine);
- high volume suction;
- rubber dam isolation (including split-dam technique) if applicable for aerosol-generating procedures;
- 3-in-1 syringe with cautions.

Besides, dentists could also consider postponing non-urgent elective dental treatments.

In addition to the standard precautions, other control measures for patients attending dental clinics, e.g., body temperature check, ask questions about COVID-19 signs and symptoms, and take traveling, occupation, contact and cluster (TOCC) history, are required to identify high-risk infectious cases and to prevent transmission of the disease in the dental setting. Patients with fever, respiratory symptoms or any relevant COVID-19 symptoms are asked to wear face masks and go for medical care immediately. Dental treatment should be limited to emergency treatment and no aerosol-generating procedures should be provided to these patients.

## India

The emergence of the COVID-19 pandemic has led to a significant impact on dentistry and the dental professionals in India where there is a very large number of dental personnel. There are almost 278,000 dentists, 81,000 under-graduate students (3rd, 4th year and interns) and 18,000 dental postgraduates involved in patient care as per the data from dental register and website of the Dental Council of India [44].

Since the emergence of the COVID-19 pandemic, various organizations such as the Indian Dental Association (IDA) [45], Dental Council of India [46] and Ministry of Health and Family Welfare [47] have issued guidelines regarding infection control and prevention of COVID-19 in dental practice. However, critical appraisal of these guidelines reveals that the majority of these are based on the general principles of infection control, and are consensus-based but without much scientific evidence. Almost all the guidelines issued in the initial period of COVID-19 pandemic have advised to limit dental work for management emergency cases only and to postpone non-emergency dental procedures.

Earlier guidelines were mainly revolving around the management of emergency cases and suspending all other dental procedures. The persistence of the pandemic has led to changes in the guidelines for the management of dental patients which are now more focused on protective measures to limit the spread of infection rather than suspending the procedures. The IDA in its most recent guidelines published in September 2020 regarding infection control and prevention of COVID-19 infection in dental clinics highlights the possible routes of transmission of infection in dental clinics and the methods to minimize the chances of exposure. The recommendations are focused on patient screening, rescheduling appointments, antiseptic mouthwash prior to clinical procedures and modifications to limit the infections while performing the dental procedures by the use of rubber dam, PPE, high speed evacuation, and autoclaving [48].

## Japan

Since the new coronavirus entered Japan, there have been two waves of newly reported cases. The peak of the second wave was passed in the 1st week of August 2020, however, the trend of infections varies between regions. Although the pandemic situation in Japan has improved from the state of emergency in April 2020, socio-economic activities have been greatly impacted. The government of Japan declared basic policies aiming to control the infection within a manageable level and to minimize the number of deaths and patients in critical conditions as well as to gradually increase the socio-economic activities. The key point of the measures is to establish the “new-lifestyle” including avoiding “3Cs” (closed spaces, crowded places and close-contact setting) and basic counter-infection measures such as keeping distance, wearing a mask and washing hands [49].

Since the COVID-19 situation in Japan is still ongoing, the Japan Dental Association (JDA) has requested dental institutions to consider the postponement of dental treatments, regular dental examinations, and house call dental services that are not urgent and where serious complications would not occur in case of such postponement [50]. According to the JDA [51], dental practice during COVID-19 situation is to strictly follow standard precautions with regards to aerosol infection. It has been recommended to use proper suction device to prevent the dispersion of droplets or aerosols emitted from the patient's mouth. Both high power suction and extraoral suction device are necessary. Moreover, a rubber dam is suggested if possible. Since the new coronavirus enters through the mucous membrane of the mouth, nose and eyes, it is necessary to wear a surgical mask and goggles or a face shield. Disinfection of dental units and the surrounding contact area by proper disinfectant, such as ≥60% alcohol or 0.05% sodium hypochlorite, for each patient is also required. Intraoral radiography for patients with a high risk of vomiting reflex, patients with asthma or respiratory illness, who are considered to be at high risk of droplets such as cough and rash should be avoided. To reduce the level of the microorganisms and to prevent infection, patients are asked to gargle with antiseptics such as povidone iodine, benzalkonium chloride before and after treatment which may be effective. Since chlorhexidine has been reported to cause anaphylactic shock in mucosal use in Japan, it has been limited to 0.05% as a mouthwash. The dental office environment should avoid the “3Cs” and basic counter-infection measures should be followed. In order to avoid crowded places and close-contact, patients are asked to come to the dental office exactly at the appointment time to minimize the number of people in the waiting room. To avoid closed spaces, the room should be well-ventilated by opening windows regularly. In addition, playground facilities and books or magazines should be removed from waiting areas to prevent contact infection. All dental care workers have to measure their body temperature twice a day. Furthermore, in case of having symptoms such as malaise, the dental staff have to report and consult the person in charge of the clinic and consider monitoring at home depending on the condition. Although dentists and dental clinic staff are at high risk of infection, there have so far been no reports in Japan of patients becoming infected through dental treatment.

## New Zealand

There have been no reported cases of cross infection from dental treatment in New Zealand (31 August 2020) [52]. The first case in New Zealand was reported on 28 February 2020. The number of new cases peaked in early April with around 89 new cases recorded per day. No new cases were reported between 22 May and 16 June 2020. New Zealand's borders were closed to non-residents on 19 March 2020, and since 10 April 2020 all returning residents have been required to undergo 2 weeks of supervised quarantine. Currently, anyone with any COVID-19 symptoms is encouraged to be tested [53]. A four-level alert level system was introduced on 21 March 2020 to manage and minimize the risk of COVID-19 in New Zealand. The system helps people to understand the current level of risk and the restrictions that legally must be followed [54]. Alert 1 (prepare) and level 2 (reduce), are in place when the disease is contained, and health and disability care services can be operated as normally as possible. Alert level 3 (restrict) and 4 (lockdown) are in place when there is high risk and it is likely the disease is not contained; healthcare services need to be reprioritized, and/or virtual, non-contact consultations are used where possible [54]. A contact tracing app has been released by the Ministry of Health to facilitate contact tracing of any new cases in New Zealand.

Oral health services are required to practice under the guidelines developed in New Zealand by the Ministry of Health and the New Zealand dental regulatory authority, the Dental Council [55]. The guidelines give detailed instructions on the oral health services that can be provided at different COVID-19 alert levels, including risk assessments, steps in assessing a patient for care, treatment requirements and so on. New rules for electronic prescriptions to support virtual care in the community were also provided from 31 March 2020. In briefly, the treatments that can be provided, appointment pre-screening, and contact tracing and PPE requirements, for low and high risk patients differ according to the alert levels. Only urgent or emergency care can be provided for patients under level 3 and level 4. For aerosol-generating procedures, patients are treated in a negative pressure room. Minimum PPE required includes N95 masks or FFP2 (filtering face piece score 2), eye protection, gloves and long sleeve impervious gown. For non-aerosol-generating procedures, a single and closed-door room is required. Minimum PPE required includes a surgical mask, eye protection, gloves and outer protective clothing as per the infection prevention and control (IPC) practice standard. Under levels 1 and 2, the dental treatment for high risk patients continues to follow the requirements of level 3 and 4. For low risk patients, both routine care as well as urgent or emergency care can be provided. The minimum PPE requirements are surgical mask, eye protection, gloves, and outer protective clothing as per the IPC practice standard. The use of closed door surgeries are encouraged wherever possible. Other measures which aim at reducing the extent and contamination of aerosol and splatter (such as use of dental dam, high volume suction and four-handed dentistry) are recommended where possible. The use of a pre-procedural mouth rinse was introduced but this requirement has subsequently been removed.

The New Zealand government and the Dental Council in New Zealand have reacted promptly during the COVID-19 pandemic and continue to do so. The alert level policy and the dental guidance system helps dental practitioners to thoroughly evaluate each patient in terms of current health status and/or contacts with potentially infected people to avoid cross-infection. Availability and effectiveness of PPE is a key determinant of the risks posed to healthcare professionals [56]. The production of aerosol and droplets during routine dental procedures contributes to the generation of highly contaminated microbial aerosol [57]; therefore management of aerosol is another important factor to be considered in the policy. There is a need for constant awareness of infectious threats that may challenge the current infection control regimen.

## Nigeria

The first case of COVID-19 was detected in Lagos, Nigeria on the 27 February 2020. The national government responded promptly by setting up a Presidential Task Force on COVID-19 on the 17 of March 2020 at which time the country had reported only 3 cases of COVID-19. By the 23 March 2020, the country shut down its airports and banned international travels in and out of the country (22 cases reported at that time) and on the 30 March 2020 it announced the lockdown of three states (Lagos, Ogun and the Federal Capital territory) with the highest number of COVID-19 cases (70 cases reported at that time). Shortly after, the entire country was locked down.

The Federal Government started to ease its lockdown measures on the 4 May 2020 following socioeconomic concerns. Nigeria is largely driven by the informal economic sector—over 83.2% of Nigerians are employed through the informal sector [58]. The lockdown therefore, meant a lot of economic hardship for the people. The palliative measures instituted during the lockdown could only reach 2.3 million of the estimated 200 million Nigerians. In view of the huge economic challenges with signals of possible social unrest, the lockdown was eased at which time the country had already recorded 2,802 cases. International flights resumed on the 20 September 2020 and schools re-opened on the 12 October 2020.

In June 2020, the Federal Ministry of Health of Nigeria issued the COVID-19 guidelines/standard operational procedures for dental practice in Nigeria. The guideline was developed in collaboration with the Nigeria Dental Association. The guidelines require that all non-emergency dental treatments should be postponed (with guidelines on considerations for dental emergencies); screening and management of staff daily for COVID-19 prior to engagement with clinical care; and screening and management of patients before and after treatment; and there are guidelines on waste management. Any staff or patient screened and showed signs and symptoms of COVID-19 are to be referred for management with the COVID-19 isolation center. Patient management includes the use of recommended PPE, mouth rinsing for 30 s using 5 ml of 1% hydrogen peroxide, avoidance of aerosol, application of rubber dam when use of high-speed handpieces and ultrasonic scaler is inevitable, and use of high-volume suction to limit aerosols. Patients are also expected to keep a scheduled appointment, maintain physical distance, maintain hand hygiene and cough etiquette. Post therapy instructions should include COVID-19 education. This policy applies to both private and public hospitals [59].

## Switzerland

The cumulative total number of laboratory-confirmed COVID-19 positive cases in Switzerland, between 24 February 2020 and 4 December 2020 were estimated to be 344,497 (313,474 cases since 8 June 2020). In this total, the laboratory-confirmed hospitalizations were reported to be 14,041 cases (10,033 cases since 8 June 2020), of which the laboratory-confirmed deaths were estimated to be around 4848 cases (3,134 cases since 8 June 2020) as updated by the Federal Office of Public Health on the 4 December 2020 [60]. The highest peak of the new cases per week was at the end of March 2020, then decreasing until the second wave of the outbreak began around mid-June 2020. Since June 2020, after the state changed from the extraordinary situation to the special situation, the form of rules, obligations, prohibitions, requirements, and recommendations generally applied for all of Switzerland, but may have differed slightly between the cantons. The community's main general rules focused on compulsory wearing of masks on public transport, including airplanes, and mandatory quarantine for travelers from a list of countries (constantly updated) with a high risk of infection. For all persons who have been in close contact with someone who has COVID-19, a quarantine of 10 working days is ordered. The SwissCovid contact tracing app was launched by the FOPH which is complementary with the conventional contact tracing methods carried out by the cantons [61].

The Association of Cantonal Dentists in Switzerland (VKZS) and the Swiss Dental Association (SSO) launched the guideline for dental practice during the COVID-19 pandemic since March 2020 [62]. The guideline is continuously revised based on the evolving pandemic situation. When it was changed, supplemented, or revoked, the new version is published. In the guideline, the quality guidelines, SSO practice hygiene (Qualitätsleitlinien SSO Praxishygiene) and the QSS of the practice (Qualitätssicherungssystem der Praxis), are emphasized to be strictly followed. Everything touched by patients or staff in the practice area must be cleaned regularly with disinfectant, for instance, hourly disinfection of all door latches and taps, seating furniture in the waiting room. If there were several dental units in a single room, adequate mutual protection must be ensured. Accompanying persons with the patient must be kept to a minimum. The appointment should be precisely on time. As far as possible, the number of patients in the waiting room is restricted to a minimum or none. The waiting time of the patients is attempted to be minimal to none. Accompanying persons are not allowed in the treatment rooms except when absolutely necessary or in special situations. To implement these restrictions, an extra 15-min is added to their regular appointment time and scheduling is synchronized such that there would be the least number of patients in the waiting area. When patients are in the waiting area, a minimum distance of at least 1.5 m between patients must be provided. Masks and hand-disinfection are mandatory. Nobody is allowed to enter the facility without a mask. Hand-disinfection is a must on entry into the facility. Hand-disinfection agents are positioned at appropriate ports within the facility. For a smooth operation of a practice, the facility must stock sufficiently the necessary protective equipment, and that they are always ensured for a minimum of at least 3 months.

Staff should take a detail patient history and ask questions related to COVID-19 symptoms. Information about close contact with COVID-19 positive people in the previous 2 weeks, quarantines, or any return from vacations in COVID-19 risk areas is collected. When entering the hospital, the patient can be given a hygienic mask to wear. If the patient's body temperature is more than 38 °C, they should be discharged and a later appointment is scheduled. When performing an aerosol-generating procedure, complete room ventilation is recommended and the air of the entire room is renewed for at least 15 min following the recommended state guidelines. When the standard protective materials (hygiene mask, treatment gloves, protective goggles, disinfectant) are not available, no treatments are carried out.

Before treatment, the patient can be instructed to gargle and rinse with mouth-rinse, such as 1.5% hydrogen peroxide (H_2_O_2_) for 30 s or povidone-iodine according to the manufacturer's instructions. The practitioner should perform treatments under a rubber dam whenever possible. If the application is not possible, other systems with a proven aerosol-reducing effect must be used. In aerosol-generating treatments without the possibility of placing a rubber dam, the treatment team should wear at least an FFP2 mask (without valve) along with other available protective equipment.

Elders aged 65 and over along with adults with the following systemic diseases: high blood pressure, chronic respiratory diseases, diabetes, immunocompromised diseases, cardiovascular diseases, cancer or obesity, are considered as high-risk groups. The dental treatment benefits must be weighed against the risk of COVID-19 infection in this high-risk group. For patients with suspected COVID-19 or a COVID-19 positive patient, only urgent emergency treatment may be carried out in a separate treatment room reserved for COVID-19 patients. They should not be in contact or mix with other patients. The patient should wear a hygienic mask immediately upon arrival at the practice and is immediately escorted to the treatment facility. The practitioners, including assistants, must at all times whilst in contact with the COVID-19 positive patient wear a FFP2 mask as well as an apron, gloves, and safety glasses. In cases where an aerosol-generating procedure is performed on such a patient, then the masks are to be worn for a further period of up to 30 min beyond the termination of the aerosol-generating procedure. Depending on the cantonal regulations, the patient can be referred for treatment at a hospital or a specialized clinic [62].

Practice staff that have suspected symptoms of COVID-19 should be tested immediately. Those whose tests are positive must quarantine at home for 10 working days from when the symptoms begin and to after 48 h when the symptoms subside. Where there is a negative result, staying at home is also mandatory until 24 h after the symptoms subside. Persons coming in contact with a COVID-19 positive individual, without a protective mask at a distance shorter than 1.5 m and for a duration of 15 min or more, are defined as a “close contact” person. The close contact persons must quarantine themselves for at least 10 days. If they have suspected symptoms during this time, they should follow the above-mentioned protocol. Other symptom-free and non-close contact staff continue to work in strict compliance with the standard measures [61].

## Thailand

Thailand had been able to control the spread of the COVID-19 rather well until the end of December 2020 when the new phase of COVID-19 transmission emerged. The total number of the cases had remained ~3,500 with < 60 deaths during the 6-month period of virtually no local transmission. However, toward the end of December 2020, a large cluster of COVID-19 cases was identified among mostly migrant workers in a seafood market close to Bangkok. New cases have since emerged in several provinces across the country. Provinces have now been categorized into four color-coded tiers according to the risk of transmission, and heightened precautionary measures have been put into place for provinces with higher risk, especially those in the red zone.

In order to control the first phase of COVID-19, the government had employed strict lockdown measures in Bangkok and other major cities from February to May 2020. The population generally cooperated well with public health measures including the use of masks, hand-washing, and social-distancing measures. Local public health volunteers country-wide are instrumental in monitoring cases and limit transmission in rural areas. These had contributed to the success in COVID-19 management in the first phase, and the combined efforts from all sectors will be needed again to control the new phase.

During the period of active local transmission (May 2020), the Department of Medical Services, Ministry of Public Health issued a joint guideline for dental practices during the pandemic, together with dental organizations, including Thai Dental Council, Dental Faculty Consortium of Thailand, Royal College of Dental Surgeons of Thailand, Private Dentist Association of Thailand, and Dental Federation of the Ministry of Public Health [63]. An adjustment to the guideline was later issued in July 2020 after a 50-day period of no local transmission to allow routine services in low risk population under standard precautions and aerosol reduction protocol [64]. The guideline provides recommendations for patient screening based on risk of exposure and signs and symptoms of COVID-19, and for patient management based on emergency and urgency of dental conditions. Screening should be performed by phone or messaging services in advance, and if possible, phone-based consultation services should be offered. In cases with high risk or possible COVID-19 infection, only emergency dental treatments should be provided, and these should be performed in an airborne infection isolation room (AIIR) or negative pressure room while using maximum PPE and source control measures. Non-emergency treatments should be postponed and high/moderate-risk patients should be referred to undergo a COVID-19 test. For non-suspected or low risk cases, emergency treatments may be provided in a separate room while using full PPE and appropriate source control measures. Non-emergency treatments may be provided in settings with good ventilation under standard precautions. An N95 or equivalent respirator should be used when an aerosol-generating procedure is necessary. COVID-19 testing is offered to health care personnel who may be exposed to possible cases, but is not routinely performed.

The guideline includes recommendations on dental clinic zoning, infection control procedures, ventilation system and PPE. It also suggests a 4-tier management protocol depending on the local and global pandemic situation, so that dental personnel can adjust their practices accordingly. The Ministry of Public Health also offered a suggested model for dental clinic ventilation system improvement, complete with a construction plan, specifications, and budget, which can be freely accessed online [65].

## Discussion

Despite the differences in the severity of the COVID-19 pandemic, the policy implications in the nine countries (Table 2) are similar in some extent as follow:

- Strict social distancing
  The COVID-19 pandemic has severely affected dental practice and dental care workers because it is a respiratory infection and can spread through aerosol and splatter. Oral healthcare practitioners (dentists, dental hygienists, therapists and dental assistants) typically work in close proximity to one another and their patients. Thus, dentists are considered to be at the highest risk of COVID-19 infection amongst all healthcare workers [66].
- Disinfection of used instruments, equipment and workspace
  Transmission of SARS-CoV-2 in the dental clinic can occur through physical proximity to the patient's face, inhalation of the virus in aerosols and splatter (through talking, coughing, sneezing, and the use of dental instruments) and/or from dental unit water [67]. The risk of transmission of SARS-CoV-2 can be significantly reduced when the working surfaces in a dental office are sufficiently cleaned and disinfected. Non-essential human contact should also be reduced. Further, bio-aerosols generated from the use of dental instruments can be appropriately managed with the appropriate disinfection of instruments and equipment, and care of the dental unit water lines.
- Strict infection control measures
  One of the observations from this review is the prompt action taken by the dental profession to reduce the risk of dental healthcare workers and their patients contracting COVID-19. The average time of release of guidelines from the day of the first report of COVID-19 in the respective country was relatively short, within 1–2 months. The action in most countries at the height of the pandemic was the closure of dental clinics to non-essential dental health services and increased conscientiousness with use of PPE. Only a small number of well-equipped dental hospitals/clinics remained open for emergency services only at the peak of the pandemic. With the easing of the strict restrictions, elective non-emergency dental care for patients with suspected or known COVID-19 was postponed for at least 2 weeks [68]. This action seemingly has helped reduce the anticipated risk of transmission of SARS-CoV-2 in dental clinics as there was only one case of dental clinic-based transmission of COVID-19 in this review of reports from nine countries. Dental teams are also very familiar with universal PPE and other cross infection control measures and thus, are likely to have been more alert to the need to evaluate their risks for infection and take appropriate actions for both their patients and themselves during this pandemic.
- Early screening and identification of possible cases
  One strategic action employed during this pandemic is the early screening and identification of possible cases of COVID-19, including asymptomatic patients who visit the dental clinic. Protocols for the triaging of patients for infected cases, including the asymptomatic patients, is a vital procedure that appears to have been effective in the management of patients before providing dental treatments under the COVID-19 pandemic situation. Further empirical studies are needed however to understand how this strategy has contributed to a reduction of risk of managing asymptomatic patients with COVID-19. National oral health policies also need evidence to support the use of screening and triaging mechanisms to reduce the risk for other infections in the dental clinic.

**Table 2:** Summary of oral health policies and guidelines during the COVID-19 pandemic in each country/region.

**Country/region**	**Screen patients**	**Personal Protective Equipment (PPE)**	**Specified requirement for clinical procedures**	**Suspend non-emergency treatment**	**Ventilation**
Canada	Yes	• SM3 masks/N95 respirators • Face shields • Disposable gloves • Long-sleeved non-sterile gowns • Scrubs • Surgical caps	• Minimizing the use of the air/water syringe • High volume evacuator • Rubber dam recommended • 4-handed dentistry for all aerosol-generating procedures	Yes	No specific requirement; recommended to contact HVAC specialist for professional evaluation of facility
China	Yes	Based on the risk classification of patients: • Low-risk patients (standard precaution): Disposable caps and gloves, surgical face masks, work clothes • Medium-risk patients (advanced protection): Disposable caps and gloves, surgical face masks or N95 face mask or equivalent, eye protection, work clothes, gowns • High-risk patients (strengthened protection): Disposable caps and gloves, N95 face mask or equivalent, eye protection, work clothes, medical protective clothes, medical isolation shoe covers	Recommend to use the following item irrespective of the patient risk level: • Rubber dam • Strong suction devices • Antiseptic mouthrinse	Yes, for patients at high or medium-risk	During the treatment, adequately ventilated room are recommended
Egypt	Yes	Yes(Mandatory according to Ministry of Health and population guideline for dental practicing during Covid-19 pandemic time)	It is recommended to limit dental practice for providing emergency dental care in the following cases: • Treatment of severe endodontic pain: Exacerbation of pulpal tissue or extraction of badly broken teeth • Treatment of deep carious lesions in deciduous teeth (children): Recommended treatment is extraction or pulpotomy • Treatment of dental abscess (swelling): Stapp incision to relief pain/extraction in case of badly broken teeth in addition to medical treatment • Treatment of dry (infected) socket: Irrigation + Palliative treatment + Medical Treatment Bringing children to dental clinic is totally prohibited except if the child seeks dental care and at this time, he/she should be accompanied by only one person (parent/guardian) Provide hand sanitizers in waiting areas/Mandatory wearing of masks and applying social distance measurements at patient waiting areas	Yes	Well-ventilated practicing areas, mainly depend on renewable fresh air with limitation of the use of air conditioner
Hong Kong	Yes	No specific requirement	Recommend to use: • Rubber dam • High volume suction • Antiseptic mouthrinse • 3-in-1 syringe with cautions	Yes	No specific requirement
India	Yes	• Non-aerosols procedures, and examination- A triple layer surgical mask and protective eyewear/face shield and gloves. • High Risk and very high-risk procedures—-N95 face masks, protective eyewear/face shields and gloves along with coverall • Moderate risks - All PPE as high risk except that the coveralls can be substituted with surgical gowns	• Preoperative mouthrinse Povidone Iodine 0.5% solution • High volume extra oral suction • Use of rubber dam is encouraged. • The 4-handed technique for controlling the infection. • The dental chair water lines should be equipped with ant retraction valves • Hand pieces with anti-retraction valves • All 3 in 1 syringe, water outlets, hand piece water pipelines, etc. should be flushed with the disinfectant solution for 30–40 s.	Yes	Ventilation and air quality management guidelines (Air circulation, strong exhaust, Maintenance of air conditioners, Indoor air cleaning system HEPA filters and UV light)
Japan	Yes	• Wear disposable gloves • Wear disposable waterproof apron • Wear face shields	• Extraoral high volume suction recommended • Isodine mouthrinse • Rubber dam recommended	No	Yes
New Zealand	Yes	• For aerosol-generating procedures: N95 masks or FFP2 (filtering face piece score 2), eye protection, gloves and long sleeve impervious gown. • For non-aerosol-generating procedures: surgical mask, eye protection, gloves and outer protective clothing as per the infection prevention and control practice standard	Reducing the extent and contamination of aerosol and splatter (such as use of dental dam, high volume suction and four-handed dentistry) are recommended.	Differ according to the alert levels	The use of closed-door surgeries are encouraged wherever possible
Nigeria	Yes	• Use long sleeve scrubs • Wear disposable gloves • Wear disposable waterproof apron over scrubs • Wear face shields • Wear N95 face masks • Wear respirators, full face shields with head cover and shoe cover for aerosol generating procedures	Recommend to use: • Rubber dam • High volume suction for aerosols • Hydrogen peroxide mouth rinse	Yes	Yes
Switzerland	Yes	In the case of aerosol-generating treatment or treatment with suspected COVID-19 patients, an FFP2 mask without a valve is recommended.	• Rubber dam whenever possible • Effective suction system • Antiseptic mouthrinse	Yes	After aerosol-generating treatment, a 15-min pause is recommended for the renewal of air ventilation.
Thailand	Yes	Recommend to use:Standard PPE (hair net, goggle, surgical mask, faceshield, disposable gloves, waterproof/isolation gown, shoe covers) or Full PPE (N95 or equivalent respirator instead of surgical mask, double disposable gloves, and leg covers in addition to standard PPE) depending on level of risk	Recommend to use: • Rubber dam • High volume suction • Antiseptic mouthrinse	Yes	Recommend: • Separation of operating and waiting areas • ventilation system improvement (no specific requirement)

The adoption of appropriate use of precautionary measures by dental health care practitioners based on the risk of exposure may have also contributed significantly to the reduced risk for COVID-19 infection in the dental clinic. In view of the fact that COVID-19 may become a continue health concern, it may be important for countries to continue the use of the various precautionary measures adopted for COVID-19 for routine dental practice. Environment and engineering controls such as air-flow management in dental practice should be strengthened to prevent nosocomial infections. So far, most of the guideline/recommendations for COVID-19 prevention for dental health care practitioners are based on the general principles of infection control. The observed low rate of infection with COVID-19 in the dental clinics may also relate to patients' actions, as some patients have stayed away from the dental clinic due to concerns about the possible risk of infection [69]. Patients who have COVID-19 like symptoms may also decide not to visit dentists because they are aware of the closure of the clinic due to patients with COVID-19 symptoms. Studies are needed to understand the factors contributing to the low infection rates in the dental clinic and the effectiveness of the various precautionary measures, as well as to determine if there are viable viral particles in saliva, aerosol, splatter and their infectivity. This will help generate evidence for infection control guidelines, which can further help mitigate the risk for other forms of infection in the dental practice.

- Postponing non-emergency treatment
  The closure of dental clinics for non-emergency dental care raises a serious concern because many patients would need regular preventive and curative services to prevent the occurrence of dental pain and inflammation. So far, little is known about the impact of the closure of the dental clinic on the oral health and well-being of these patients. A strategic action taken by a number of dentists in the countries reviewed is the use of teledentistry, namely online consultation, offered to public by dental professionals through social media so that preliminary evaluation and suggestions on oral health problems and information are available for the public. The effectiveness of teledental practice needs to be evaluated as this is a potential oral health management strategy that can be adopted in the long run and become institutionalized as an oral health practice in the national oral health policy. Studies can help improve this practice, and reduce the risk for dental practice due to a standstill.
- Improving ventilation system
  It is indeed challenging for dental professionals to adapt to new norms caused by the COVID-19 pandemic. Clinics have had to redesign their physical and work space to improve the ventilation system and to limit aerosol generation during dental procedures. The cost implications and the size of the clinics may imply that these changes will take place at different paces for different clinics. New facility requirements and workspace designs may be adopted for the long term and gradually become a common feature for dental practices around the world. As politicians in the United States said, never waste a good crisis. This COVID-19 public health crisis has become an opportunity for dentistry to reform and to move toward less invasive and more preventive approaches where need dictates [70].

The COVID-19 pandemic has, however, exacerbated socioeconomic and ethnic inequalities [71], and may also have magnified health inequalities [72]. Rural places and hard-to-reach communities that had access to oral health care through outreach and sponsored programs have had services suspended due to various reasons such as travel restriction and social distancing. This sadly increases the inequality in access to oral health care among these vulnerable populations. Vulnerability to oral health inequalities may also be further entrenched due to the likely increased cost in dental practice resulting from the adoption of stringent infection control practices. The cost increase may be significant enough to keep many persons away from seeking dental care if they have little financial resources to pay directly for the dental service. This raises the need to advocate for the universal health coverage plans to be accessible for vulnerable and marginalized communities, and also for access to oral health care services not being suspended due to crisis like the COVID-19 pandemic.

There are limitations of this review. First, the number of countries and regions included in this study is small, although we tried to invite collaborators from different places around the world so as to cover the global situation. Still, it may not represent the global profile since the included countries and regions are from a convenient sample of collaborators who were purposefully invited and agreed to participate in the study. Despite this, findings from different geographical areas included in this review are consistent. Second, the current review only provides a summary of the situation up to the end of the year 2020; the information should be updated based on the constantly changing situation. As the COVID-19 pandemic is ongoing worldwide, oral health policies and guidelines may need to be updated accordingly. Further studies are necessary to formulate evidence-based oral health guidelines and policies to balance treatment needs and the risk of spreading COVID-19.

## Conclusion

To conclude, despite the differences in the severity of the COVID-19 pandemic in the selected counties/regions, the changes in oral health policies and guidelines are consistent to some extent. These include the suspension of non-emergency dental care services at the peak of the outbreak, strict infection control practices, reductions in aerosol production during dental procedures, improvement of the air quality in dental clinics, and maintenance of social distancing.

## Author Contributions

CJ, DD, CC, and EL contributed to the design of the work and revised the paper critically. All authors contributed to the data extraction, analysis and interpretation, drafted the paper, and approved the final version of the manuscript to be published.

## Conflict of Interest

The authors declare that the research was conducted in the absence of any commercial or financial relationships that could be construed as a potential conflict of interest.
